# Synovial fluid viscosity with synovial fluid cell count, valuable diagnostic marker of prosthetic joint infections

**DOI:** 10.1038/s41598-025-00760-6

**Published:** 2025-05-09

**Authors:** Samo Roškar, René Mihalič, Anže Mihelič, Rihard Trebše

**Affiliations:** 1 Valdoltra Orthopaedic Hospital, Jadranska Cesta 31, 6280 Ankaran, Slovenia; 2 Faculty of Medicine, University of Ljubljana, Zaloška Cesta 9, 1000 Ljubljana, Slovenia

**Keywords:** Synovial fluid viscosity, Synovial fluid cell count, Total joint arthroplasty, Prosthetic joint infections, Medical research, Diagnostic markers, Orthopaedics

## Abstract

In the absence of standard criteria for prosthetic joint infections (PJI), several diagnostic modalities have been proposed mostly concentrating on novel biochemical markers. The physical chemistry markers received scarce attention. Synovial fluid (SF) viscosity could be considered as marker for PJI, however, its diagnostic value of PJI remains unknown. Our study aimed to determine the potential of SF viscosity as a diagnostic marker of PJI and compare it to SF cell count with differential (CCD). We prospectively analysed 123 SF samples (58 septic and 65 aseptic) for viscosity and CCD of SF obtained during hip and knee revision procedures. The diagnosis of PJI based on EBJIS criteria. The viscosity cut-off for PJI was calculated and the diagnostic power was compared to CCD. The mean SF viscosity in the PJI group was 8.5 ± 0.4 mPa s and 103.2 ± 18.8 mPa s in the aseptic group (*p* < 0.05). SF viscosity achieved 100% sensitivity and 85.3% specificity, with AUC 0.832 (95% CI 0.739, 0.925). Combination of SF viscosity and CCD achieved AUC 0.951 (95% CI 0.919, 0.987). SF viscosity is more sensitive but slightly less specific in diagnosing PJI than SF CCD. Best diagnostic value is achieved combining SF viscosity with CCD in detection of PJI.

## Introduction

Total hip and knee arthroplasty are considered a highly successful treatment of advanced osteoarthritis^1–3^. Regarding pain relief and functional outcome, total hip arthroplasty (THA) has been considered a surgery of the twenty-first century^3^. Thus, in Western Europe until 2040 the incidence of THA and total knee arthroplasty (TKA) is expected to grow by 23% and 45%, respectively^2^. In the US, the projected increase in the incidence of primary TKA is 43% between 2020 and 2050^1^. Despite the success of primary THA and TKA and the gradual decrease of the implant-related causes for revision procedures, prosthetic joint infection (PJI) related revision rates are expected to grow as much as 176% for THA and 170% for TKA by 2030^4^. The predicted growth of PJI revision cases is projected to create a substantial economic burden on the healthcare systems^5^. PJI has the reported rate of around 1% after total hip arthroplasty and up to 2% after total knee arthroplasty^6^. PJI is the most common reason for the revision procedure after TKA and the third most common reason after THA^7,8^. Furthermore, there is an increasing evidence that up to 20% of aseptic failures are low-grade PJI^9,10^. Since the treatment of PJI fundamentally differs from aseptic failures, it is essential to perform accurate diagnostic. The treatment of missed PJI as aseptic failure often leads to multiple revisions with poor functional outcome and high mortality rates^11^. Additionally, patients revised for PJI have substantially higher in-hospital mortality rate than patients being revised due to aseptic reasons^12,13^. The estimated 5-year mortality for THA and TKA PJI is 15% and 25%, respectively, and is even higher than mortality for breast cancer, melanoma, and non-Hodgkin lymphoma^5^. Despite the importance of the early and correct diagnosis of PJI, to date there is no single test to reliably diagnose all PJIs^14,15^. In recent years a great advance has been achieved with formulation of PJI diagnostic criteria proposed by Musculoskeletal Infection Society (MSIS)/International Consensus Meeting (ICM), Infectious Diseases Society of America (IDSA), and European Bone and Joint Infection Society (EBJIS)^14,16,17^. IDSA criteria are concentrating mainly on combination of clinical presentation and intraoperative diagnostics while excluding inflammatory markers as diagnostic criterion^17^. MSIS/ICM criteria were introduced in 2011 and were the first criteria using scoring system with the “major” (presence of sinus tract, and at least two positive cultures) and “minor” (elevated C-reactive protein (CRP), elevated synovial leukocyte count, elevated % of synovial neutrophils, presence of pus fluid, only one positive intraoperative culture, intraoperative frozen sections histology) criteria^18^. The MSIS/ICM criteria were further revised in 2013 with introduction of leukocyte esterase in synovial fluid as additional minor criteria and in 2018 additionally α-defensin in synovial fluid and D-dimer in serum were added to minor criteria^16,19^. The EBJIS criteria were introduced in 2021 and they are considered to be most sensitive among all criteria available^17,20^. In recent years, most studies have concentrated on novel biochemical markers for PJI with calprotectin, d-lactate, interleukin (IL)-6, and α-defensin being studied most^21–25^. None of the biochemical markers tested has shown any significant added diagnostic value compared to synovial fluid cell count with differential (CCD)^11,14^. In contrast, the potential physical chemistry markers received scarce attention. Studies of Mazzucco et al. emphasized the existing differences in viscosity between normal, osteoarthritic, and rheumatic synovial fluid^26^. Galandáková et al. have shown the difference in synovial fluid (SF) viscosity between the patients with osteoarthrosis and those with aseptic loosening^27^. Mazzucco et al. and Fu et al. suggested that SF viscosity could be considered as an important marker for PJI^26,28^. However, the diagnostic value of the SF viscosity for PJI remains unknown. The aim of our study was to determine if SF viscosity is a more reliable diagnostic marker of PJI than the SF CCD.

## Methods

**Fig. 1:**
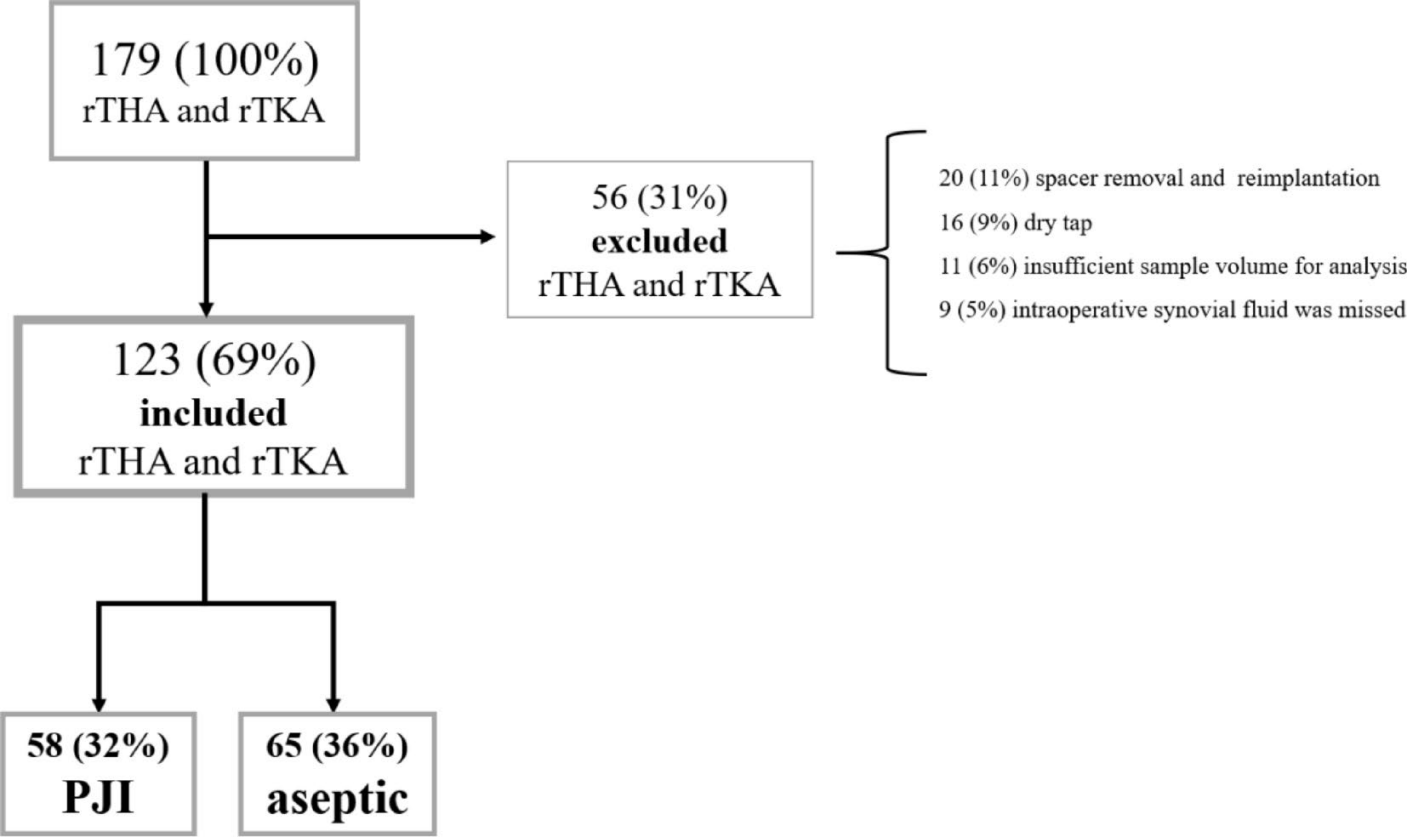
Flowchart diagram showing the number of patients excluded based on each criterion. *rTHA* revision total hip arthroplasty, *rTKA* revision total knee arthroplasty, *PJI* periprosthetic joint infection.

In the present study, we conducted a single-centre, prospective cohort study to investigate the diagnostic value of SF viscosity in differentiation between septic and aseptic total joint arthroplasty (TJA) failure. All samples were collected at Valdoltra Orthopaedic Hospital between January 2021 and January 2023, we prospectively included and analysed SF samples obtained during total hip and knee revision surgeries. In this time period altogether 179 revision surgeries of THA and TKA were performed. The aspiration of SF used for the study was performed before joint capsule incision inside laminar flow operating theatre during the revision surgery. All revisions were performed by one of the three senior consultants. No patient was intentionally excluded from the study. Therefore, the only reason a patient was not included in the study was a dry tap (16 cases) or inadequate sample volume obtained during intraoperative joint aspiration with exposed but not open capsule (11 cases) and in 9 cases the intraoperative synovial fluid aspiration was unintentionally missed during the surgery. Thus, 123 patients (69% of all THA and TKA revision cases in this time period) were finally included in the study. None of the patients was missed from the follow-up. The detailed data about number of cases in each category are presented on Fig. 1. The patient demographics, sex, age, body mass index (BMI), American Society of Anaesthesiologists (ASA) score, and infection location werecollected from medical records.

**Fig. 2:**
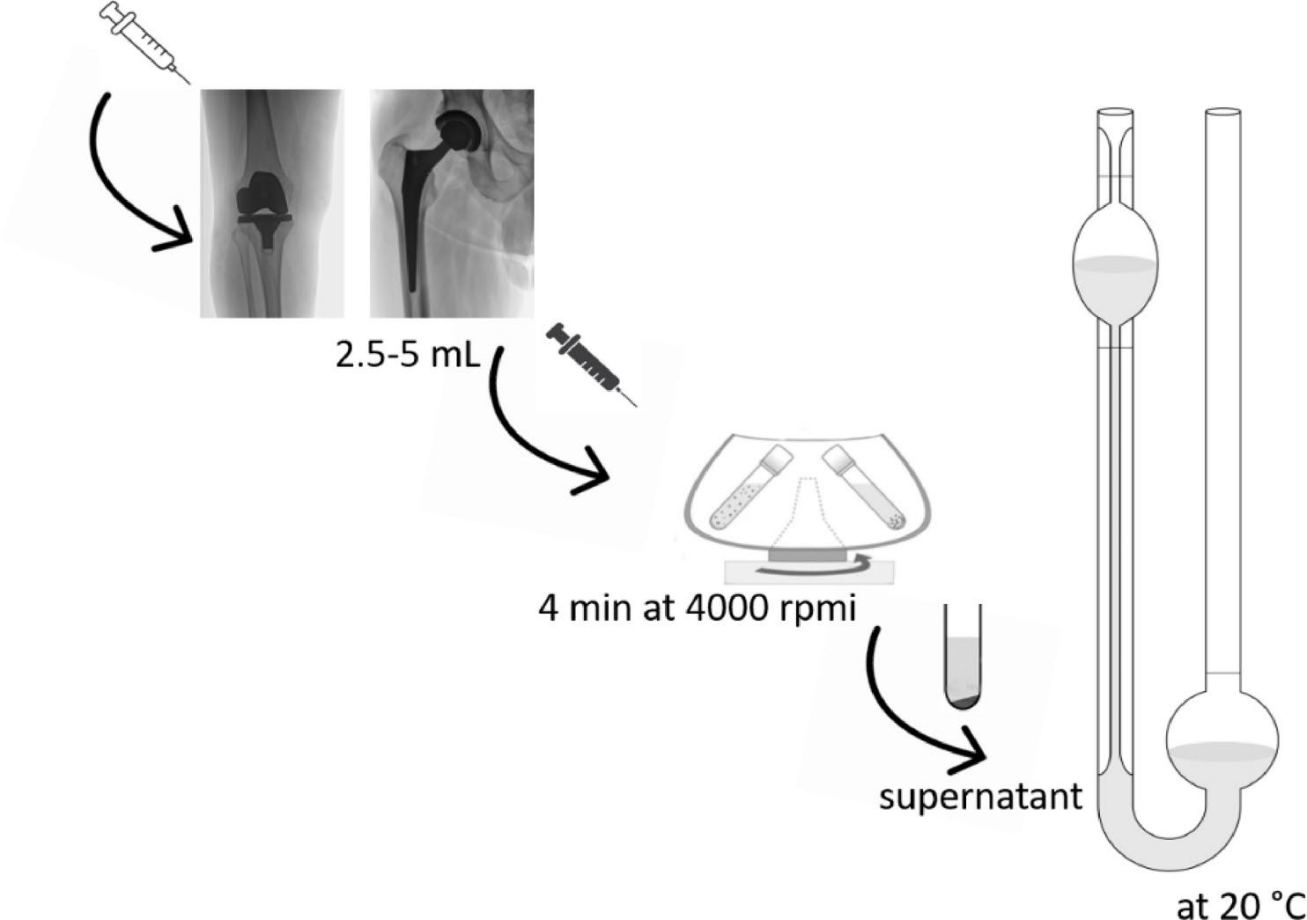
Schematic presentation of synovial fluid analysis for viscosity.

**Fig. 3:**
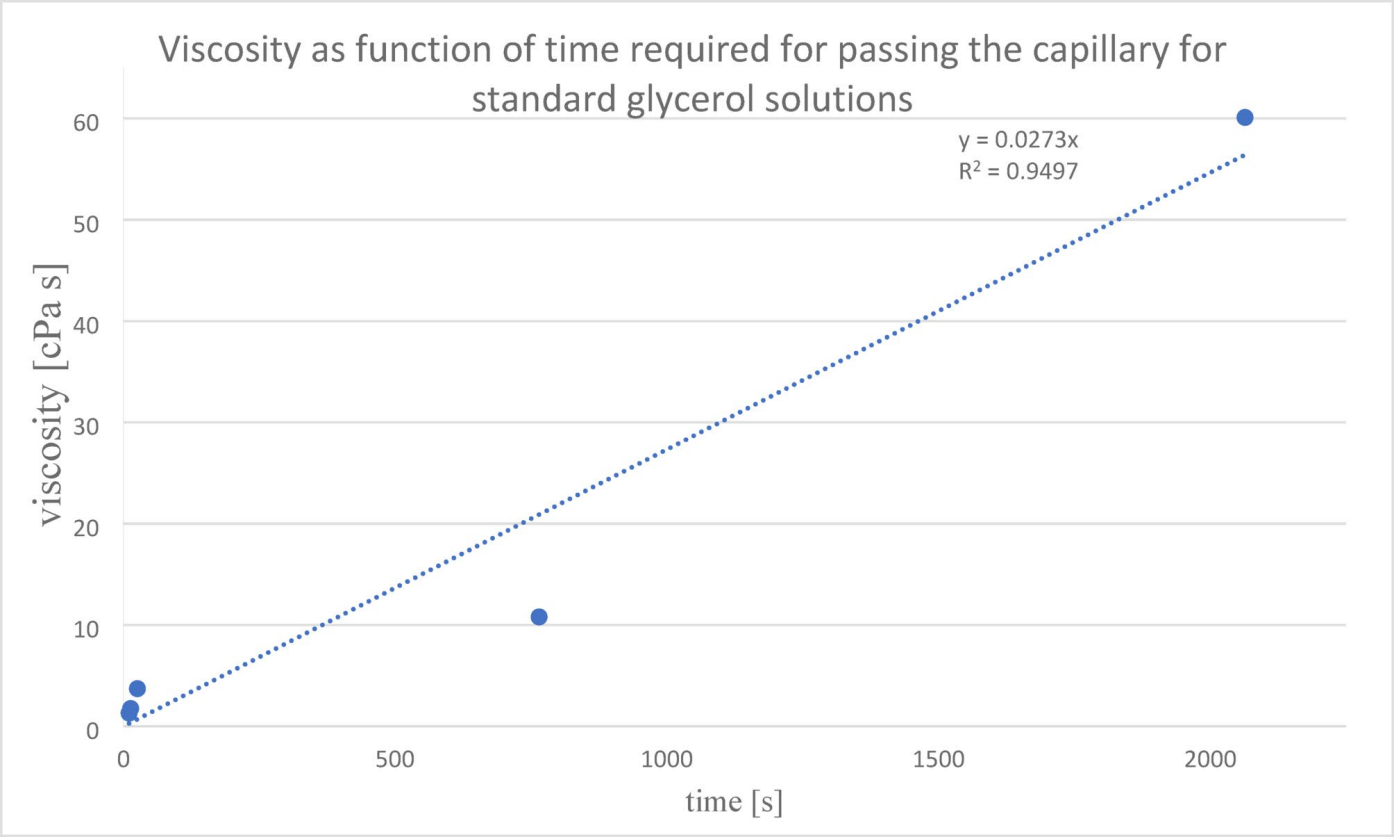
Viscometer calibration curve with standard glycerol solutions.

We sampled 2.5–5 mL of SF for viscosity and CCD measurements. Intraoperatively, 1 mL of the sample was analysed for the CCD. The remaining SF was analysed for viscosity. For SF viscosity measurement, the sample was centrifuged for 4 min at 4000 rpm. The supernatant was transferred to Ostwald viscometer and the time required to pass the viscometer at 20 °C was determined. The viscosity is directly proportional to the time required for a certain volume of the fluid to pass the capillary and can be expressed as shown by Eq. (1). The whole process of the SF samples analysis for viscosity is presented in Fig. 2. During each surgery at least 5 microbiological and multiple histopathological samples were harvested, and explant sonication was performed. The microbiological cultures were grown in the laboratory until definite microbiological result for at least 14 days. As a gold standard diagnostic protocol, the definite diagnosis of septic or aseptic failure, was based on the EBJIS definition criteria. For PJI diagnosis to be confirmed microbiologically, growth of the same microbe has to be obtained in at least two tissue cultures or one tissue culture and sonication fluid or only sonication fluid with > 50 CFU/mL. For calculation of viscosity from the time measures according to Eq. (1), standard glycerol solutions were used by mass percentage of 10, 20, 40, 60, 80%. Glycerol standard solutions were prepared and the reference viscosity values were used as provided by Segur and Oberstar^29^. Measuring sedimentation time for each solution, the calibration curve was obtained (Fig. 3) using reported standard viscosities provided by Segur and Oberstar^29^. For our viscometer, the threshold sedimentation time for detecting PJI was set at 65 s and using standard glycerol solutions, the threshold viscosity for PJI was calculated to be 17.7 mPa s.

1 $$\text{Viscosity }\left(\upeta \right)=\frac{\pi p{r}^{4}}{8Vl}\cdot \text{time}\left(\text{t}\right)$$

where η is viscosity, *p* is hydrostatic pressure, r is radius of the capillary, l is length of the capillary, V is volume of the liquid, and t is time required od a certain volume to pass the capillary.

Statistical data analysis was performed with IBM SPSS Statistics 23.0 for Windows (IBM Corp. Armonk, USA). Continuous numerical variables were expressed as mean ± standard deviation. Categorical variables were expressed as number of cases and the corresponding percentage. Analysing basic characteristics of septic and aseptic subgroup Student’s t-test was carried out to compare significant differences when numeric variable was measured, χ^2^ test was used when nominal categorical variables were compared and Mann–Whitney test was applied for ordinal categorical variables. *P* values < 0.05 were considered as statistically significant difference. Receiver operating characteristic (ROC) curves and the area under the curve (AUC) were used to measure diagnostic values of SF viscosity and CCD. The optimal predictive cut-off for viscosity was determined using the Youden index. Binomial logistic regression was used to analyse the diagnostic value of SF viscosity and CCD.

The study was approved by Valdoltra Orthopaedic Hospital institutional ethical board (ID no. 08-2023). All procedures were performed in studies involving human participants, and were in accordance with the ethical standards of the institutional ethical committee and with the 1964 Helsinki declaration and its later amendments or comparable ethical standards. Informed consent was obtained from all subjects and/or their legal guardian(s).

## Results

In the time period 179 THA and TKA revisions were performed. Fifty-six (31%) revisions were either a dry tap or inadequate sample volume (obtained volume less than 2.5 mL) to perform examination of both, SF viscosity and SF CCD. Thus, 123 (69%) revision TJAs (75 knees and 48 hips) were included in the study. Further demographical data with comparison between the PJI and the aseptic group age, BMI, ASA, the joint involved, serum leukocytes and serum CRP on admission are presented in Table 1. Differences in demographic data between the two groups had no statistical significance.

**Table 1:** Basic characteristic of patients included in the study.

Patients	PJI	Aseptic	*p* value
Number (n (%))	58 (47%)	65 (53%)	
Age (mean ± SD) (year)	71.2 ± 9.9	69.4 ± 9.5	0.31
Gender (n (%))			0.59
Male	26 (45%)	26 (40%)	
Female	32 (55%)	39 (60%)	
BMI (mean ± SD) (kg/m^2^)	30.4 ± 6.5	30.1 ± 5.8	0.79
Joint (n (%))			0.22
Hip	26 (45%)	22 (34%)	
Knee	32 (55%)	43 (66%)	
ASA (n (%))			0.56
I	0	0	
II	21 (36%)	26 (40%)	
III	31 (53%)	35 (54%)	
IV	6 (11%)	4 (6%)	
Lkc at admission (mean ± SD) (× 10^9^)	7.7 ± 3.96	6.6 ± 3.09	0.08
CRP at admission (mean ± SD) (mg/L)	25 ± 105.2	5 ± 7.44	0.12

*ASA* American Society of Anaesthesiologists Score, *BMI* body mass index, *Lkc* leucocytes, *CRP* C-reactive protein.

**Fig. 4:**
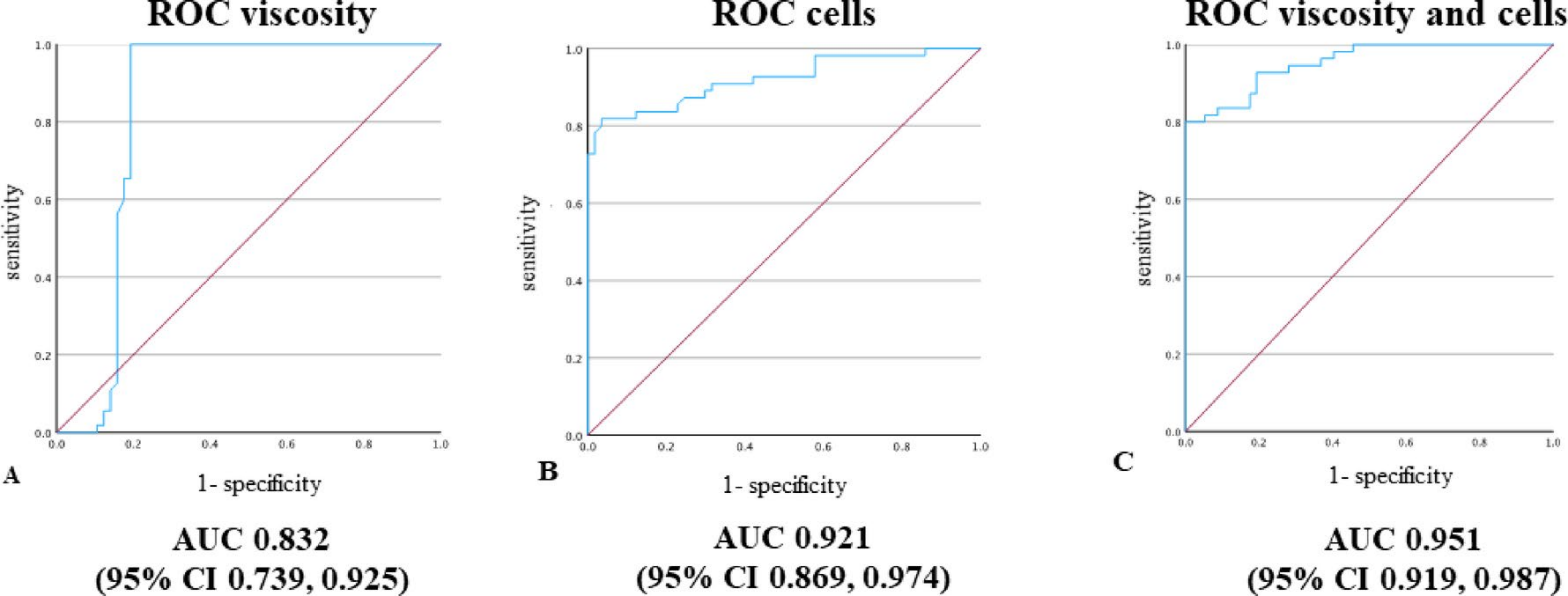
(**A**) Receiver operating characteristic (ROC) curve for synovial fluid (SF) viscosity. (**B**) ROC curve for SF cell count with differential (CCD). (**C**) ROC curve for SF viscosity and CCD.

Appling EBJIS diagnostic criteria, there were 58 septic and 65 aseptic diagnoses. The value of synovial fluid viscosity was significantly higher in the aseptic group compared with the PJI group (Table 2). The viscosity threshold for detecting PJI was set at 65 s. Considering the calibration curve obtained with measurement of standard glycerol solutions and applying the Eq. (1), the threshold viscosity was determined to be 17.7 mPa s. Mean SF viscosity in the PJI group was 8.5 ± 0.4 mPa s and 103.2 ± 18.8 mPa s in the aseptic group (*p* < 0.05). As a diagnostic marker of PJI, SF viscosity achieved 100% sensitivity and 85.3% specificity, with area under the curve (AUC) of 0.853 (95% CI 0.772, 0.937) (Fig. 4a). When setting the diagnostic threshold at infection likely category according to the EBJIS criteria, at 1.5 10^9^/L, the specificity and sensitivity of SF CCD were 98.2% and 78.2%, respectively, with AUC of 0.921 (95% CI 0.869, 0.974) (Fig. 4b). Together, SF viscosity and CCD achieved a combined AUC of 0.951 (95% CI 0.919, 0.987) (Fig. 4c). Detailed data on specificity and sensitivity of SF CCD at different diagnostic threshold values according to EBJIS criteria are presented in Table 3.

**Table 2:** Difference in values of traditional plasma, SF markers and SF viscosity between PJI in aseptic group.

	PJI		Aseptic	
	Mean ± SD	95% CI	Mean ± SD	95% CI
SF leukocytes (cells 10^9^)	30.0 ± 5.9	18.2–41.8	0.47 ± 0.078	0.32–0.63
% of SF PMN (mg/L)	83 ± 3	78–88	16 ± 3	10–21
Viscosity of SF (mPa s)	8.5 ± 0.4	7.6–9.3	103.2 ± 18.8	70.4–140.9

*SF* synovial fluid, *PJI* periprosthetic joint infection, *SD* standard deviation, *95% CI* 95% confidence interval, *PMN* polymorphonuclear cells.

**Table 3:** Sensitivity and specificity of synovial fluid cell count and synovial fluid differential at infection likely and infection confirmed threshold according to EBJIS criteria^6^.

	Threshold	Sensitivity (mean, 95% CI)	Specificity (mean, 95% CI)	PPV (mean, 95% CI)	NPV (mean, 95% CI)
Synovial fluid cell count (10^9^/L)					
Inf. likely	1.5	78.2 72.8–83.6	98.2 92.8–100	97.8 92.4–100	83.1 77.7–88.5
Inf. conf.	3	70.7 65.4–76.0	95.4 90.1–100	93.1 87.3–98.4	78.5 73.2–83.8
Synovial fluid differential (% of PMN cells)					
Inf. likely	65	87.9 85.5–90.3	93.7 91.3–97.3	91.1 88.7–93.5	89.6 87.2–92.0
Inf. conf.	85	72.4 70.0–74.8	100 97.6–100	100 97.6–100	80.2 77.8–82.6
Viscosity (mPa s)	17.7	100 91.8–100	85.3 77.1–93.5	85.3 77.1–93.5	100 91.8–100

*Inf. likely* infection likely category according to EBJIS criteria, *Inf. conf.* infection confirmed category according to EBJIS criteria, *PMN* polymorphonuclear, *PPV* positive predictive value, *NPV* negative predictive value.

Detailed microbiological results of PJIs are presented in Table 4. Of the 10 cases incorrectly diagnosed as aseptic based on SF viscosity, 2 were acute trauma with joint haematoma and 8 had metallosis. The SF CCD in all these cases was < 0.5 10^9^/L. Of the 12 cases incorrectly diagnosed as aseptic based on SF CCD, 6 cases were culture negative, 4 were *C. acnes* and 2 were *S. epidermidis* isolates in microbiology.

**Table 4:** Results of microbiological examination for 58 patients considered as PJI.

Result of microbiological examination	n (%)
*Staphylococcus aureus*	9 (16%)
*Staphylococcus epidermidis*	7 (12%)
*Staphylococcus lugdunensis*	3 (5%)
*Streptococcus agalactiae*	2 (3%)
*Streptococcus gallolyticus*	1 (2%)
*Streptococcus oralis*	1 (2%)
*Streptococcus pyogenes*	1 (2%)
*Corynebacterium jeikeium*	1 (2%)
*Corynebacterium striatum*	1 (2%)
*Cutibacterium acnes*	5 (9%)
*Serratia marscescens*	3 (5%)
*Escherichia coli*	1 (2%)
*Enterobacter cloacae*	1 (2%)
Mixed flora	7 (12%)
Culture negative	15 (26%)

## Discussion

To our knowledge, this is the first study to evaluate the role of SF viscosity measurement in TJA revision surgery and to compare SF viscosity and SF CCD as diagnostic test for PJI. Our study shows, that SF viscosity is more sensitive, but less specific for PJI detection compared to SF CCD. The combination of SF viscosity and SF CCD was proven to have high diagnostic accuracy for intraoperative decision making.

Despite high recent scientific activity in the field of the diagnostic criteria, the diagnosis of PJI remains challenging, especially in low-grade infections^14,20,30^. In recent years, lots of studies have been published dealing with biochemical markers of PJI^21–25^. In general, all known biochemical markers for detection of PJI can be divided in two major groups, direct and indirect. As indirect markers of PJI, we can consider the molecules associated with immune system response (interleukins, calprotectin, leukocyte-esterase, α-defensin, …) and the direct markers representing the molecules related to bacteria and their metabolism (d-lactate). Among PJI biochemical markers mainly the indirect ones have been studied most (calprotectin, IL-6 and α-defensin, leukocyte-esterase), while d-lactate being the only widely studied direct biomarker^21–25^. Calprotectin in different studies showed the sensitivity range from 79.1 to 99.9% and specificity from 83.5 to 100%^31^. Different interleukins had sensitivity from 41.9 to 99.8% and specificity from 38.6 to 96.4%^32^. α-Defensin had sensitivity from 54 to 100% and specificity from 89 to 99%^33,34^. Leukocyte-esterase showed sensitivity between 30.1 and 62.8%, and specificity between 83.5 and 98.7%^31^. d-lactate had sensitivity from 75 to 95% and specificity from 73 to 88%^35^. Recently, Schindler et al. published a systematic review on novel diagnostic markers for PJI, they found 19 different studies, examining different parameters in synovial fluid from proteins, molecules, exosomes, DNA, interleukins and lysosomes^11^. However, in the past 5 years none of the analysed biomarkers has outperformed the accuracy of SF cell count with differential probably because all of them intrinsically measure the amount of active immune response in the synovial fluid^11,36^. According to recent review of Sigmund et al., all biochemical markers available to date failed when applied for diagnostics of low-grade PJI^36^. In cases of low-grade PJI even conventional biomarkers such as CRP, serum leukocytes, CCD are inconclusive diagnostic tools^11,14,20,36–38^.

In contrast, the potential physical chemistry markers received limited attention. Viscosity is one of the features connected with the rheological properties of SF. Main constituents of SF related to greater viscosity are different kinds of polymeric molecules present in normal SF, e.g. hyaluronic acid, lipids and proteins^39^. Some studies dealing with rheological properties demonstrated that the SF viscosity was lowered with decreasing concentration of mainly proteins and hyaluronic acid^26,40^. Studies of Mazzucco et al. emphasized the existing differences in viscosity between normal, osteoarthritic, and rheumatic SF^26^. Galandáková et al. have shown in their study difference in SF viscosity between the patients with osteoarthritis and those with aseptic loosening^27^. Mazzucco et al. and Fu et al. suggest that SF viscosity could be considered as an important marker for PJI^26,28^. With our study, we have shown that viscosity for PJI group was 8.5 ± 0.4 mPa s and 103.2 ± 18.8 mPa s for PJI and aseptic group, respectively. The SF viscosity determined for PJI group in our study is similar to the viscosity reported for infected cases reported by Fu et al.^28^. While in our study, we observed higher SF viscosity in the aseptic group compared to the reported values by Fu et al.^28^. Possible explanation for this observation could be the reason that we included in the aseptic group also four cases of partial knee endoprostheses which is by findings of Mazzucco et al. who have shown that SF viscosity relates to the degree of preserved cartilage in the joint^26^. Moreover, the observed viscosity in our aseptic group is still in the range of SF viscosity reported by Mazzucco et al.^26^.

Up to date, according to EBJIS diagnostic criteria, SF CCD with differential is considered the most reliable for pre/intraoperative decision making about presence of PJI^14^. Several studies have shown that SF CCD is often negative in low-grade infections^30,37^. SF CCD has 78.2% (95% CI 72.8–83.6) sensitivity and 98.2 (95% CI 92.8–100) specificity at threshold value 1.5 × 10^9^ cells/L, for infection likely category within EBJIS criteria. With our study, we have demonstrated that SF viscosity exhibit 100% with (95% CI 91.8–100) sensitivity and 85.3% with (95% CI 77.1–93.5) specificity for PJI. Based on present study results, SF viscosity is more sensitive for PJI than SF CCD. Taken together, SF viscosity combined with SF CCD have AUC 0.951 (95% CI 0.919–0.987). Thus, the combination of both appears to be the optimal diagnostic procedure for intraoperative decision making about presence of PJI. Considering our results, where 12 cases incorrectly diagnosed as aseptic based on SF CCD having the SF viscosity measurement in range of PJI, on microbiological examination most abundant microbes were *C. acnes* and *S. epidermidis*. In PJIs due to *C. acnes*, we noticed that in 4 out of 5 (80%) cases SF CCD was negative while SF viscosity all of them was in range of PJI. Similarly, we obtained SF viscosity values in range of PJI in 2 out of 7 (29%) cases of *S. epidermidis* PJIs with negative SF CCD. Considering culture negative infections, we had 6 out of 15 (40%) cases having negative SF CCD while SF viscosity was in range for PJI. On the basis of our samples we can speculate, that SF viscosity outperforms SF CCD with differential in low-grade infections. However, further research of SF viscosity is still required on greater cohorts of low-grade infections.

From methodological point of view, the presented method of SF viscosity measurement could be optimised using novel viscometers with smaller required sample volume. With smaller SF sample volume further research is required to analyse also the SF viscosity of total shoulder, ankle and elbow endoprostheses. Furthermore, additional research is still required to analyse the exact influence of metallosis and crystallopathies on SF viscosity. From data analysis point of view, the main limitation of present study is absence of the “gold standard” method for PJI diagnostics to which we could compare novel diagnostics methods.

## Conclusions

Our results demonstrate that SF viscosity is more sensitive but slightly less specific for PJI detection than SF CCD. The study also demonstrates the diagnostic value of combining SF viscosity with CCD in decision making in TJA revision surgery. We propose to use SF viscosity measurement in combination with standard SF CCD to improve intraoperative diagnostics of PJI. Establishing SF viscosity and CCD as point of care test has the possibility to become a fast, reliable, and affordable intraoperative selection of appropriate surgical modality particularly in patient with preoperative diagnostic uncertainty.

## Data Availability

All raw data related to the study are freely available to any researcher for non-commercial purposes, without breaching participant confidentiality by the corresponding author on reasonable demand.

## References

[1] Klug, A. et al. The projected volume of primary and revision total knee arthroplasty will place an immense burden on future health care systems over the next 30 years. *Knee Surg. Sports Traumatol. Arthrosc.* **29**(10), 3287–3298 (2021). 32671435 10.1007/s00167-020-06154-7 PMC7362328

[2] Rupp, M. et al. Projections of primary TKA and THA in Germany from 2016 through 2040. *Clin. Orthop. Relat. Res.* **478**(7), 1622–1633 (2020). 32168057 10.1097/CORR.0000000000001214 PMC7310374

[3] Learmonth, I. D. et al. The operation of the century: Total hip replacement. *Lancet* **370**(9597), 1508–1519 (2007). 17964352 10.1016/S0140-6736(07)60457-7

[4] Schwartz, A. M. et al. Projections and epidemiology of revision hip and knee arthroplasty in the United States to 2030. *J. Arthroplasty* **35**(6S), S79–S85 (2020). 32151524 10.1016/j.arth.2020.02.030 PMC7239745

[5] Premkumar, A. et al. Projected economic burden of periprosthetic joint infection of the hip and knee in the United States. *J. Arthroplasty* **36**(5), 1484–1489 (2021). 33422392 10.1016/j.arth.2020.12.005

[6] Jin, X. et al. Estimating incidence rates of periprosthetic joint infection after hip and knee arthroplasty for osteoarthritis using linked registry and administrative health data. *Bone Jt. J.* **104-B**(9), 1060–1066 (2022). 10.1302/0301-620X.104B9.BJJ-2022-0116.R1 PMC9948458 36047015

[7] Koh, C. K. et al. Periprosthetic joint infection is the main cause of failure for modern knee arthroplasty: An analysis of 11,134 knees. *Clin. Orthop. Relat. Res.* **475**(9), 2194–2201 (2017). 28573549 10.1007/s11999-017-5396-4 PMC5539036

[8] Melvin, J. S. et al. Early failures in total hip arthroplasty—A changing paradigm. *J. Arthroplasty* **29**(6), 1285–1288 (2014). 24444568 10.1016/j.arth.2013.12.024

[9] Jacobs, A. M. E. et al. The unsuspected prosthetic joint infection: Incidence and consequences of positive intra-operative cultures in presumed aseptic knee and hip revisions. *Bone Jt. J.* **99-B**(11), 1482–1489 (2017). 10.1302/0301-620X.99B11.BJJ-2016-0655.R2 29092987

[10] Hipfl, C. et al. Unexpected low-grade infections in revision hip arthroplasty for aseptic loosening: A single-institution experience of 274 hips. *Bone Jt. J.* **103-B**(6), 1070–1077 (2021). 10.1302/0301-620X.103B6.BJJ-2020-2002.R1 34058865

[11] Schindler, M. et al. Novel diagnostic markers for periprosthetic joint infection: A systematic review. *Front. Cell. Infect. Microbiol.* **13**, 1210345 (2023). 37529352 10.3389/fcimb.2023.1210345 PMC10388554

[12] Reinhard, J. et al. In-hospital mortality of patients with periprosthetic joint infection. *Bone Jt. Open* **5**(4), 367–373 (2024). 38663864 10.1302/2633-1462.54.BJO-2023-0162.R1 PMC11045279

[13] Szymski, D. et al. Comparison of mortality rate and septic and aseptic revisions in total hip arthroplasties for osteoarthritis and femoral neck fracture: An analysis of the German Arthroplasty Registry. *J. Orthop. Traumatol.* **24**(1), 29 (2023). 37329492 10.1186/s10195-023-00711-9 PMC10276794

[14] McNally, M. et al. The EBJIS definition of periprosthetic joint infection. *Bone Jt. J.* **103-B**(1), 18–25 (2021). 10.1302/0301-620X.103B1.BJJ-2020-1381.R1 PMC7954183 33380199

[15] Trebse, R. & Roskar, S. Evaluation and interpretation of prosthetic joint infection diagnostic investigations. *Int. Orthop.* **45**(4), 847–855 (2021). 33555351 10.1007/s00264-021-04958-x

[16] Parvizi, J. et al. The 2018 definition of periprosthetic hip and knee infection: An evidence-based and validated criterion. *J. Arthroplasty* **33**(5), 1309–1314 (2018). 29551303 10.1016/j.arth.2018.02.078

[17] Osmon, D. R. et al. Diagnosis and management of prosthetic joint infection: Clinical practice guidelines by the Infectious Diseases Society of America. *Clin. Infect. Dis.* **56**(1), e1–e25 (2013). 23223583 10.1093/cid/cis803

[18] Parvizi, J. et al. Definition of periprosthetic joint infection: Is there a consensus?. *Clin. Orthop. Relat. Res.* **469**(11), 3022–3030 (2011). 21751038 10.1007/s11999-011-1971-2 PMC3183198

[19] Shohat, N. et al. Development and validation of an evidence-based algorithm for diagnosing periprosthetic joint infection. *J. Arthroplasty* **34**(11), 2730–2736 (2019). 31279603 10.1016/j.arth.2019.06.016

[20] Sigmund, I. K. et al. Diagnostic accuracy of neutrophil counts in histopathological tissue analysis in periprosthetic joint infection using the ICM, IDSA, and EBJIS criteria. *Bone Jt. Res.* **10**(8), 536–547 (2021). 10.1302/2046-3758.108.BJR-2021-0058.R1 PMC8414440 34409845

[21] Vale, J. S. et al. Synovial fluid biomarkers for the diagnosis of periprosthetic joint infection—A systematic review and meta-analysis of their diagnostic accuracy according to different definitions. *J. Arthroplasty* **38**(12), 2731–2738 (2023). 37321521 10.1016/j.arth.2023.06.017

[22] Karbysheva, S. et al. Synovial fluid d-lactate—A novel pathogen-specific biomarker for the diagnosis of periprosthetic joint infection. *J. Arthroplasty* **35**(8), 2223–2229 (2020). 32269008 10.1016/j.arth.2020.03.016

[23] Yang, F. et al. Plasma fibrinogen in the diagnosis of periprosthetic joint infection. *Sci. Rep.* **11**(1), 677 (2021). 33436902 10.1038/s41598-020-80547-z PMC7803950

[24] Gehrke, T. et al. The accuracy of the alpha defensin lateral flow device for diagnosis of periprosthetic joint infection: Comparison with a gold standard. *J. Bone Jt. Surg. Am.* **100**(1), 42–48 (2018). 10.2106/JBJS.16.01522 29298259

[25] Mihalič, R. et al. Synovial fluid interleukin-6 is not superior to cell count and differential in the detection of periprosthetic joint infection. *Bone Jt. Open* **1**(12), 737–742 (2020). 33367280 10.1302/2633-1462.112.BJO-2020-0166.R1 PMC7750741

[26] Mazzucco, D. *Variation in Joint Fluid Composition and Its Effect on the Tribology of Replacement Joint Articulation*. PhD thesis. Massachusetts (MIT, 2003).

[27] Galandáková, A. et al. Characteristics of synovial fluid required for optimization of lubrication fluid for biotribological experiments. *J. Biomed. Mater. Res. B Appl. Biomater.* **105**(6), 1422–1431 (2017). 27086677 10.1002/jbm.b.33663

[28] Fu, J. et al. Synovial fluid viscosity test is promising for the diagnosis of periprosthetic joint infection. *J. Arthroplasty* **34**(6), 1197–1200 (2019). 30837099 10.1016/j.arth.2019.02.009

[29] Segur, J. B. & Oberstar, H. E. Viscosity of glycerol and its aqueous solutions. *Ind. Eng. Chem.* **43**(9), 2117–2120 (1951).

[30] Renz, N. et al. Orthopedic implant-associated infections caused by *Cutibacterium* spp.—A remaining diagnostic challenge. *PLoS ONE* **13**(8), e0202639 (2018). 30125299 10.1371/journal.pone.0202639 PMC6101412

[31] Grassi, M. et al. Synovial biomarkers to detect chronic periprosthetic joint infection: A pilot study to compare calprotectin rapid test, calprotectin ELISA immunoassay and leukocyte esterase test. *J. Arthroplasty* **37**(4), 781–786 (2022). 34998909 10.1016/j.arth.2021.12.040

[32] Fröschen, F. S. et al. Analysis of synovial biomarkers with a multiplex protein microarray in patients with PJI undergoing revision arthroplasty of the hip or knee joint. *Arch. Orthop. Trauma Surg.* **140**, 1883–1890 (2020). 32133538 10.1007/s00402-020-03388-5

[33] Renz, N. et al. Alpha defensin lateral flow test for diagnosis of periprosthetic joint infection: Not a screening but a confirmatory test. *J. Bone Jt. Surg. Am.* **100**, 742–750 (2018). 10.2106/JBJS.17.01005 29715222

[34] Balato, G. et al. Laboratory-based versus qualitative assessment of α-defensin in periprosthetic hip and knee infections: A systematic review and meta-analysis. *Arch. Orthop. Trauma Surg.* **140**, 293–301 (2020). 31300864 10.1007/s00402-019-03232-5

[35] Yermak, K. et al. Performance of synovial fluid d-lactate for the diagnosis of periprosthetic joint infection: A prospective observational study. *J. Infect.* **79**, 123–129 (2019). 31125637 10.1016/j.jinf.2019.05.015

[36] Sigmund, I. K. et al. Serum inflammatory biomarkers in the diagnosis of periprosthetic joint infections. *Biomedicines* **9**, 1128 (2021). 34572314 10.3390/biomedicines9091128 PMC8467465

[37] Ottink, K. D. et al. Factors to consider when assessing the diagnostic accuracy of synovial leukocyte count in periprosthetic joint infection. *J. Bone Jt. Infect.* **4**(4), 167–173 (2019). 31555502 10.7150/jbji.34854 PMC6757010

[38] Brumat, P. et al. Clinical and laboratory predictors for prosthetic joint infection within the first postoperative days following primary total hip and knee arthroplasty. *Int. Orthop.* **47**(9), 2173–2179 (2023). 37421426 10.1007/s00264-023-05891-x PMC10439017

[39] Park, J. B. et al. Role of hyaluronic acid and phospholipid in the lubrication of a cobalt–chromium head for total hip arthroplasty. *Biointerphases* **9**(3), 031007 (2014). 25280848 10.1116/1.4886255

[40] Mathieu, P. et al. Rheologic behavior of osteoarthritic synovial fluid after addition of hyaluronic acid: A pilot study. *Clin. Orthop. Relat. Res.* **467**(11), 3002–3009 (2009). 19418104 10.1007/s11999-009-0867-x PMC2758976

